# Anodal and Cathodal tDCS Over the Right Frontal Eye Fields Impacts Spatial Probability Processing Differently in Pro- and Anti-saccades

**DOI:** 10.3389/fnins.2018.00421

**Published:** 2018-06-27

**Authors:** Philip Tseng, Mu-Chen Wang, Yu-Hui Lo, Chi-Hung Juan

**Affiliations:** ^1^Graduate Institute of Humanities in Medicine, Taipei Medical University, Taipei, Taiwan; ^2^Research Center of Brain and Consciousness, Taipei Medical University, Taipei, Taiwan; ^3^Shuang-Ho Hospital, Taipei Medical University, Taipei, Taiwan; ^4^Institute of Cognitive Neuroscience, National Central University, Taoyuan, Taiwan; ^5^Brain Research Center, National Central University, Taoyuan, Taiwan

**Keywords:** brain stimulation, tDCS, tACS, state-dependence, homeostatic tDCS, location probability

## Abstract

Learning regularities that exist in the environment can help the visual system achieve optimal efficiency while reducing computational burden. Using a pro- and anti-saccade task, studies have shown that probabilistic information regarding spatial locations can be a strong modulator of frontal eye fields (FEF) activities and consequently alter saccadic behavior. One recent study has also shown that FEF activities can be modulated by transcranial direct current stimulation, where anodal tDCS facilitated prosaccades but cathodal tDCS prolonged antisaccades. These studies together suggest that location probability and tDCS can both alter FEF activities and oculomotor performance, yet how these two modulators interact with each other remains unclear. In this study, we applied anodal or cathodal tDCS over right FEF, and participants performed an interleaved pro- and anti-saccade task. Location probability was manipulated in prosaccade trials but not antisaccade trials. We observed that anodal tDCS over rFEF facilitated prosaccdes toward low-probability locations but not to high-probability locations; whereas cathodal tDCS facilitated antisaccades away from the high-probability location (i.e., same location as the low-probability locations in prosaccades). These observed effects were specific to rFEF as tDCS over the SEF in a separate control experiment did not yield similar patterns. These effects were also more pronounced in low-performers who had slower saccade reaction time. Together, we conclude that (1) the overlapping spatial endpoint between prosaccades (i.e., toward low-probability location) and antisaccades (i.e., away from high-probability location) possibly suggest an endpoint-selective mechanism within right FEF, (2) anodal tDCS and location probability cannot be combined to produce a bigger facilitative effect, and (3) anodal rFEF tDCS works best on low-performers who had slower saccade reaction time. These observations are consistent with the homeostasis account of tDCS effect and FEF functioning.

## Introduction

Learning regularities that exist in the environment can help the visual system achieve optimal efficiency while reducing processing load. This is true from foraging behavior in the wild (Allman, 2000) to knowing where to look in scene perception (Gegenfurtner, 2016) or even sports (Land and McLeod, 2000). Studies have shown that the processing of probabilistic information can be implicit (Chun and Jiang, 1998; Fiser and Aslin, 2001; Geng and Behrmann, 2005; Tseng et al., 2011), and is reflected in the efficiency by which it guides one's eye movements toward the probable locations (e.g., Peterson and Kramer, 2001). As such, studies that have employed eye movement tasks have repeatedly shown that targets in the high probability locations are detected faster than targets that appear in less likely locations (Miller, 1988; Carpenter and Williams, 1995; Geng and Behrmann, 2005; Gmeindl et al., 2005; Milstein and Dorris, 2007, 2011; Summerfield and Egner, 2009).

### Probabilities and pro- and anti-saccades

To investigate how the visual system processes probabilistic information, eye movement task involving pro- and anti-saccade is an excellent candidate because of its simplicity, and well-delineated cognitive architecture and neurophysiological basis (Schall and Hanes, 1993; Basso and Wurtz, 1997; Carpenter, 1999; Schall and Thompson, 1999; Schall, 2001, 2004, 2009; Schiller and Kendall, 2004). In this task, prosaccades are eye movements toward a cue, while antisaccades are eye movements moving away from the cue. The visual attention and oculomotor system is capable of learning probabilistic information in the environment, and subsequently predicting where a target of interest may be located. Consequently, prosaccades toward targets in the high probability locations are faster than targets in other locations (e.g., Miller, 1988; Geng and Behrmann, 2005; Liu et al., 2010).

In a typical pro- and anti-saccade task, it is frequently observed that antisaccades have longer latencies than prosaccades—a delay known as the antisaccade cost (Hallett, 1978). The difference between pro- and anti-saccade latency reflects the operation of different cognitive components. Everling and Fischer (1998) have suggested that the suppression of automatic response (i.e., prosaccade) and the generation of saccade toward the opposite direction are both important sub-processes that contribute to a successful antisaccade. This highlights the possible competing nature between pro- and anti-saccades (Kristjánsson et al., 2001, 2004; Kristjánsson, 2007). Importantly, the factor of location probability, when introduced in this paradigm, has been shown to be powerful enough to modulate the magnitude of the antisaccade cost (Liu et al., 2010). In one of our previous studies, Liu et al. (2010) used a probability orienting task that had 75% target probability in one location, and 25% evenly distributed in the remaining three locations. Antisaccade trials were interleaved but did not embed any spatial probability components. We found faster prosaccades and no effect in antisaccades, but only in the high probability location. Therefore, in that experimental context, probabilistic information was able to modulate saccadic behaviors in a way that was prosaccade-specific and location-specific.

### Neural mechanisms

Studies in monkeys have demonstrated that the frontal eye fields (FEF) is involved in at least two stages of oculomotor control in the context of a saccade task: visual selection (see Thompson et al., 1996, 1997; Bichot and Schall, 1999) and motor preparation (see Juan et al., 2004, 2008). Evidence has also suggested that the supplementary eye field (SEF) can be crucial to oculomotor learning (Chen and Wise, 1995), reward prediction and detection (Amador et al., 2000; Uchida et al., 2007), performance monitoring (Stuphorn et al., 2000), and antisaccades (Schlag-Rey et al., 1997; Munoz and Everling, 2004). To clarify the contributions of FEF and SEF in processing probabilities information within the oculomotor system, we have previously conducted a TMS study that selectively impaired the functioning of either FEF or SEF (Liu et al., 2010). When coupled with theta burst TMS that interfered with rFEF activities, we found prolonged saccade latencies in both saccade types (i.e., a general oculomotor function). We also observed slower prosaccades only toward the high-probability location, while the same TMS protocol did not produce any effect when applied over the SEF. Similarly, in another TMS study, Juan et al. (2008) demonstrated that FEF is critically involved in the pro- and anti-saccade task in two distinct time windows, including the visual attentional processing stage and the saccade preparation stage. Both of these stages are important functions now associated with the FEF. Based on these TMS findings, if anodal and cathodal tDCS can modulate probabilistic information processing in an excitatory and inhibitory manner, respectively, then the straightforward prediction is that prosaccade would be faster toward the high-probability (and possible low-probability as well) locations, and slower to the same locations after cathodal tDCS.

Although the probability paradigm has not been used in combination with tDCS to date, one important study by Kanai et al. (2012) did investigate an equal-probability version of this saccade task, with anodal or cathodal tDCS over the FEF. These authors found that anodal tDCS facilitated prosaccades, whereas cathodal tDCS prolonged antisaccades. These results seem to suggest a dissociation between anodal and cathodal stimulation in terms of the types of saccades they impact (anodal for pro and cathodal for anti), as well as the direction of their effect (facilitating vs. slowing).

### The current study

Kanai et al.'s study (Kanai et al., 2012) clearly demonstrated tDCS as a modulator of oculomotor behavior. However, since location probability has been shown to be a strong modulator of eye movements both at the behavioral level (Geng and Behrmann, 2005; Liu et al., 2010) and neurophysiological level (Dorris and Munoz, 1998; Schall, 2004; Liu et al., 2011), precisely how these two modulating factors may interact with each other remains unclear. In the present study, we used a similar setup as those used by Kanai et al. (2012) and Liu et al. (2010, 2011) and applied anodal or cathodal tDCS over rFEF. To control for current flow in the brain, we included a control experiment of anodal tDCS over SEF in addition to the two within-subject sham-tDCS sessions.

## Methods

### Participants

Sixty participants (31 male, 29 female) between the age of 20 and 30 were recruited from National Central University. Participants were randomly assigned into 3 experiments: the anode experiment (*n* = 20), cathode experiment (*n* = 20), and the control experiment (*n* = 20). Participants' data were incorporated into group data for further analysis only if they showed a positive probability effect during practice (Liu et al., 2010). This criterion was used because, to investigate the potential interaction between location probability and tDCS, we had to ensure that our participants were actually sensitive and responsive to the manipulation of location probability. This left us with 20 participants in the anode condition, 15 in the cathode condition, and 18 in the control experiment. All participants had normal or correct-to-normal vision, and received monetary payment for their participation upon completion of the experiment. All subjects gave written informed consent in accordance with the Declaration of Helsinki, and the study protocol was approved by the Institutional Review Board of the Linkou Chang Gung Memorial Hospital, Taoyuan City, Taiwan.

### Apparatus

Participants sat in a dimly lit room with their head stabilized with a chinrest. Stimuli were presented on a 19-inch color cathode ray tube monitor, positioned 86 cm in front of the participants The monitor had a resolution of 1,024 × 768 pixels and a vertical refresh rate of 100 Hz. The brightness of stimuli and background were measured by a ColorCAL colorimeter (Cambridge research systems). Eye movements were recorded from the left eye with an EyeLink II tracker (SR Research Ltd.) and the task was programmed in Experimental Builder (SR Research Ltd). The sampling rate was set at 500 Hz. Anodal and cathodal stimulation was delivered via a NeuroConn DC-STIMULATOR PLUS (neuroCare Group) and a pair of electrodes housed in 4 × 4 cm saline-soaked sponge coverings. The FEF electrode was secured in position using an elastic headband, whereas the reference electrode was placed over the left cheek and secured by medical tapes. The peak intensity was set at 1.5 mA, which lasted for 10 min plus a 15 s ramp-up and ramp-down time.

### Task and procedures

**Figure 1:**
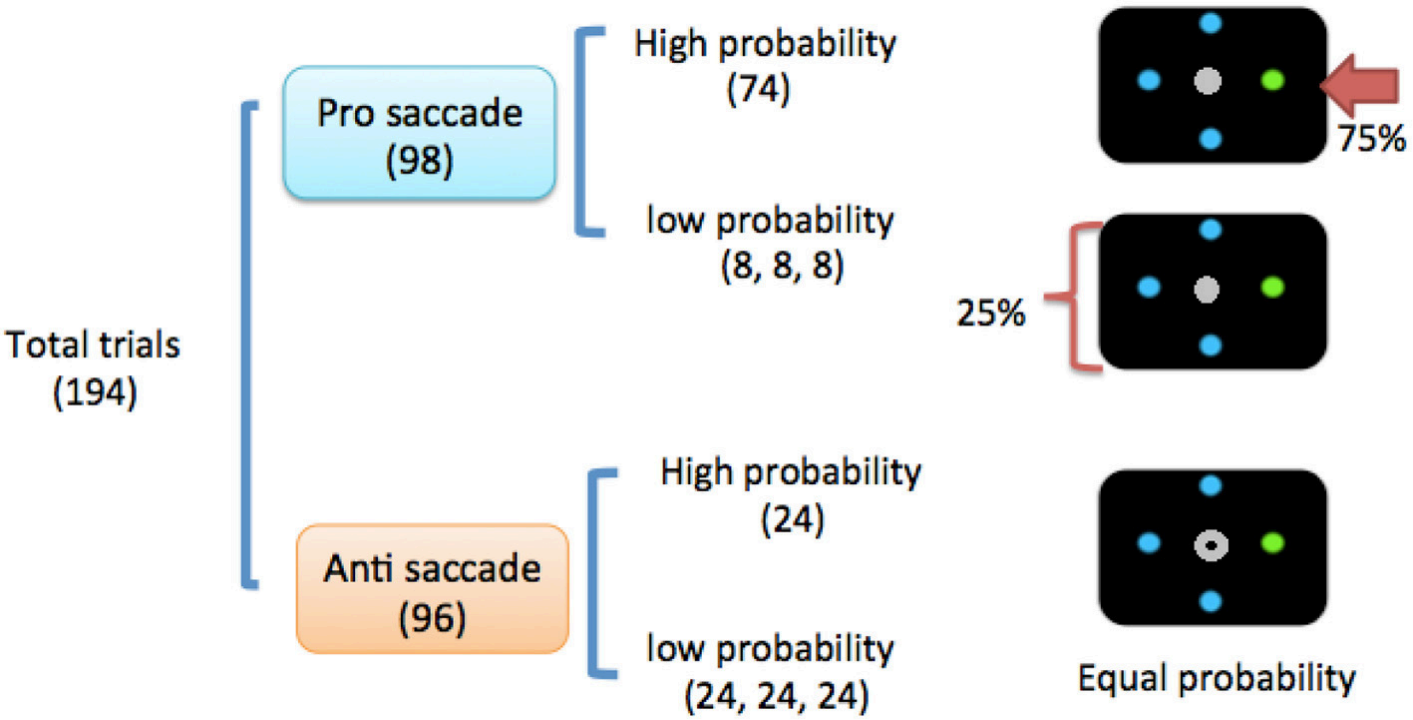
Task design. The numbers in the parentheses denote the number of trials and relative probability. Note that location probability was only manipulated in prosaccade trials, and not antisaccade trials. In the prosaccade trials, the high-probability location would only occur at either the left or right location (horizontal) location, and would not occur at a vertical location. Antisaccades have equal probabilities at all 4 locations (24 trials per location).

The experiment consisted of 2 sessions (sham vs. active tDCS) that were counterbalanced across participants. The 2 sessions were at least 1 week apart to avoid any unanticipated carryover effects. Each session contained 194 trials with roughly equal pairings of prosaccade and antisaccade probabilities (prosaccade: 98 trials; antisaccade: 96 trials, see Figure 1). Location probability was only manipulated in prosaccade trials.

In each session, 75% of prosaccade trials were to be made to the high-probability location (right) and the remaining 25% of trials were evenly distributed over the other 3 low-probability locations (left, top, and down; Figure 1). All trials were interleaved in random order regardless of saccade type (prosaccade vs. antisaccade) or probability (low vs. high), which ensured that the programming of a prosaccade or antisaccade had to be withheld until participants had finished analyzing the central saccadic indicator (Olk and Kingstone, 2003; Juan et al., 2008; Jóhannesson et al., 2013).

In this study we employed a pro- and anti-saccade paradigm that we have previously used with TMS stimulation (Liu et al., 2010, 2011) with slight modifications. An eye-tracker calibration procedure was performed at the beginning of the experiment. After calibration, participants performed 40 practice trials and then the formal experimental sessions. Participants were instructed to perform the task as quickly and accurately as possible. Each trial started when participants fixated at the central fixation cross (0.5°) for 400 ms. A search display consisted of a saccadic indicator in the center, and 4 stimuli in the periphery, would then appear (Figure 1).

In the search display, the saccadic indicator (circles 2°) was always in the center and the 4 circles were 6.5° from the fixation cross at the left, right, top, and bottom positions. There were 2 versions of the singleton: blue (Commission International de l'Eclairage, *x* = 0.18, *y* = 0.19) among green (*x* = 0.27, *y* = 0.51) circles or green among blue circles. Colors in the array were approximately isoluminant (blue 27.7 cd/m^2^, green 28.4 cd/m2) and presented on a black background. Participants were instructed to make a single saccade according to the saccadic indicator. The saccadic indicator consisted of two kinds of circles. If a solid circle was presented, participants were to perform a prosaccade to the colored singleton. And if a hollow circle was presented, an antisaccade away from the colored singleton and to the opposite side was required.

In the original paradigm (Liu et al., 2010, 2011), the contrast brightness of two concentric circles was 40.1 and 32.6 cd/m^2^, and the saccade latencies of pro- and anti-saccades were 665.4 ms and 724.3 ms, respectively. In order to shorten saccade latencies and reduce cognitive load during the discrimination process, the saccade indicators were modified into a solid circle versus a hollow circle. This modification was expected to reduce saccade latencies and ensure that the modulation of saccade latencies was caused by the probabilistic information. Upon completion of each trial, the computer displayed “Next” at the center of the monitor. Participants could either rest or press the space bar to continue onto the next trial at any time.

### tDCS protocol

In both sessions, participants received tDCS (sham vs. active) before performing the search task. The order of the sham and active sessions was counterbalanced across all participants, and the sessions were at least 24 h apart. This study used a single-blind design, where the participants were naïve to the purpose of the experiment and were not aware of which stimulation session they were participating in.

In both sessions, the center of the tDCS electrode was placed over the target site (rFEF or SEF), with the reference electrode placed over the left cheek to avoid confounding cortical activations (Nitsche and Paulus, 2001; Nitsche et al., 2003a,b; Im et al., 2012). The sites for rFEF and SEF stimulation were based on previous TMS studies that used a similar paradigm (Ro et al., 2002; Juan et al., 2008; Liu et al., 2011). In these TMS studies, FEF was functionally localized using a simple saccade task during which TMS was delivered for 500 ms at 10 Hz over candidate sites anterior to the hand motor area in the right hemisphere using a grid of points separated from each other by 1 cm. The site that resulted in the longest saccade latencies was marked as the target point for stimulation during the main task. We computed the mean rFEF and SEF coordinates from the subject pool of our previous study (Liu et al., 2011), and derived the steps (in cm) from the vertex that would reach and cover these mean coordinates in these participants. This gave us 3 cm anterior and 5 cm lateral from the vertex for rFEF localization, and 3 cm anterior and 0.5 cm lateral from the vertex for SEF localization.

The current was applied for 10 min, plus a 15 s ramp-up and ramp-down time, with the peak intensity at 1.5 mA. These parameters are well within the most recent recommendations on tDCS safety, proposed by (Antal et al., 2017) (< 4 mA and up to 60 min duration per day) and by Bikson et al., 2016, 2017 (≤4 mA and ≤40 min in one day). In the sham condition, tDCS was turned on for the 15 s ramp-up and 15 s ramp-down time, with 0 s duration in between.

After the stimulation, participants rested for 5 min before participating in the formal experiment. Each session lasted about 20 min such that the experiment length did not exceed the expected duration of the tDCS effect (Stagg and Nitsche, 2011).

### Data analysis

Accuracy and SRT data were analyzed with a repeated-measures 2 × 2 × 2 × 2 ANOVA with within-subject factors of saccade type (prosaccad vs. antisaccade), location probability (high vs. low), stimulation (sham vs. active), and between-subject factor of polarity (anodal vs. cathodal).

Saccades were identified when the velocity and acceleration of eye movements exceeded 30°/s and 8,000°/s^2^, respectively. Only the first saccade made by the participant on each trial was collected. Eye movements were recorded as correct saccades when the eye landed within a computer-defined square boundary (2° × 2°, not visible to the participants) centered on the target. Saccade latency was defined as the time interval between target onset and the initiation of a saccade. To identify outliers, a boxplot method was used to find data that were either 1.5 times the interquartile (subtracting the first quartile from the third quartile) lower than the first quartile, or 1.5 times the interquartile range higher than the third quartile.

The screening criterion for group analysis was whether the participant showed a positive probability effect or not. This positive effect was defined as the mean SRT of prosaccade made to low probability minus the mean SRT of prosaccade to high probability locations, regardless of effect size.

## Results

Mean percent correct rate for the anodal tDCS group was 78.5% (pro = 82.7%, anti = 74.1%) in the sham session and 79.9% (pro = 80.4%, anti = 79.4%) in the active session; for the cathodal group it was 81.0% (pro = 86.4%, anti = 75.4%) in the sham session and 80.4% (pro = 85.7%, anti = 75.1%) in the active session (Supplementary Table 1). Incorrect trials were not included in the analysis of SRT data.

Accuracy data were analyzed with a repeated-measures 2 × 2 × 2 × 2 ANOVA with within-subject factors of saccade type (prosaccad vs. antisaccade), location probability (high vs. low), stimulation (sham vs. active), and between-subject factor of polarity (anodal vs. cathodal). There was a significant main effect of saccade type [*F*_(1, 33)_ = 14.546, *p* = 0.001, η^2^_*p*_ = 0.306] and location probability [*F*_(1, 33)_ = 30.155, *p* < 0.001], but no effect pertaining to stimulation [*F*_(1, 33)_ = 0.807, *p* = 0.375, η^2^_*p*_ = 0.024] or polarity [*F*_(1, 33)_ = 0.044, *p* = 0.836, η^2^_*p*_ = 0.001]. In terms of interactions, the 4-way interaction was not significant [*F*_(1, 33)_ = 0.030, *p* = 0.863, η^2^_*p*_ = 0.001], or any of the 3-way interactions [saccade type × stimulation × polarity: *F*_(1, 33)_ = 1.391, *p* = 0.248, η^2^_*p*_ = 0.040; location probability × stimulation × polarity: *F*_(1, 33)_ = 0.025, *p* = 0.876, η^2^_*p*_ = 0.001; saccade type × location probability × polarity: *F*_(1, 33)_ = 0.017, *p* = 0.898, η^2^_*p*_ = 0.001; location probability × stimulation × saccade type: *F*_(1, 33)_ = 0.190, *p* = 0.666, η^2^_*p*_ = 0.006]. There was a significant 2-way interaction between saccade type and location probability [*F*_(1, 33)_ = 32.859, *p* < 0.001, η^2^_*p*_ = 0.499], stimulation and saccade type [*F*_(1, 33)_ = 7.255, *p* = 0.011, η^2^_*p*_ = 0.180], and saccade type and polarity [*F*_(1, 33)_ = 6.885, *p* = 0.013, η^2^_*p*_ = 0.173]. Everything else was not statistically significant [stimulation × polarity: *F*_(1, 33)_ = 0.007, *p* = 0.936, η^2^_*p*_ < 0.001; location probability × polarity: *F*_(1, 33)_ = 0.458, *p* = 0.504, η^2^_*p*_ = 0.014; location probability × stimulation: *F*_(1, 33)_ = 1.480, *p* = 0.232, η^2^_*p*_ = 0.043]. Therefore, accuracy data seem to be not affected by the presence of tDCS.

Saccade latencies were analyzed with a repeated measures 2 × 2 × 2 × 2 ANOVA with within-subject factors of saccade type (prosaccad vs. antisaccade), location probability (high vs. low), stimulation (sham vs. active), and between-subject factor of polarity (anodal vs cathodal). There was a main effect of stimulation [*F*_(1, 33)_ = 5.528, *p* = 0.025, η^2^_*p*_ = 0.143], saccade type [*F*_(1, 33)_ = 48.475, *p* < 0.001, η^2^_*p*_ = 0.595], and location probability [*F*_(1, 33)_ = 9.951, *p* = 0.003, η^2^_*p*_ = 0.232], and no main effect for polarity [*F*_(1, 33)_ = 0.195, *p* = 0.662, η^2^_*p*_ = 0.006]. We observed a significant 4-way interaction [*F*_(1, 33)_ = 4.873, *p* = 0.034, η^2^_*p*_ = 0.129], as well as 3-way interactions between saccade type, stimulation and polarity [*F*_(1, 33)_ = 11.888, *p* = 0.002, η^2^_*p*_ = 0.265], and between saccade type, location probability, and stimulation [*F*_(1, 33)_ = 7.491, *p* = 0.010, η^2^_*p*_ = 0.185]. We also observed significant 2-way interactions between saccade type and location probability [*F*_(1, 33)_ = 99.336, *p* < 0.001, η^2^_*p*_ = 0.751], and between polarity and location probability [*F*_(1, 33)_ = 6.347, *p* = 0.017, η^2^_*p*_ = 0.161]. All other two-way and three-way interactions were not statistically significant.

### Anodal FEF tDCS

**Figure 2:**
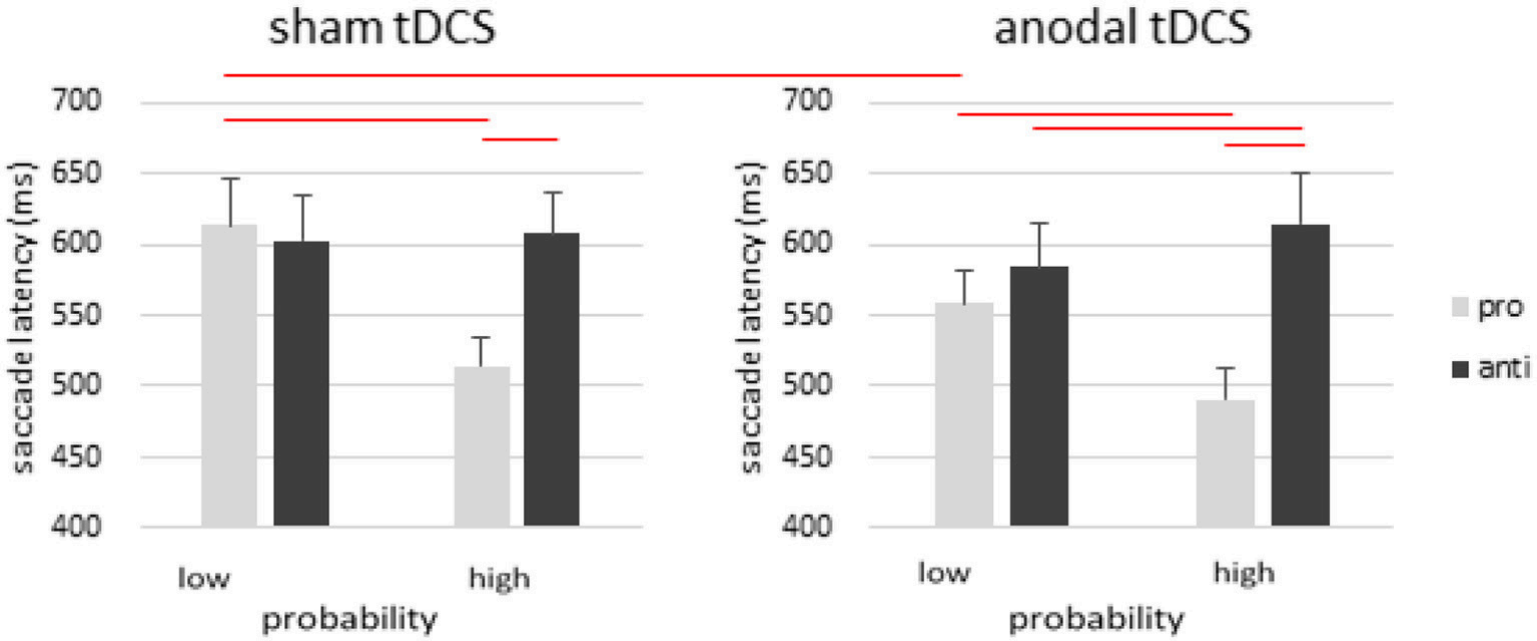
Effects of anodal tDCS on rFEF. There was a robust effect of location probability in prosaccades, as well as a pro- and anti-saccade tradeoff in the high probability condition such that antisaccades became slower even though probability information was only manipulated in prosaccades. Anodal tDCS over rFEF facilitated prosaccades toward the low-probability locations but not the high-probability location. Error bars represent standard error of the mean.

To further explore the 4-way interaction in saccade latency, we conducted a 3-way ANOVA with saccade type, location probability, and stimulation as within-subject factors in the anodal and cathodal group (Figure 2). In the anodal group, there was a significant main effect of saccade type [*F*_(1, 19)_ = 21.326, *p* < 0.001, η^2^_*p*_ = 0.529] and probability [*F*_(1, 19)_ = 21.461, *p* < 0.001, η^2^_*p*_ = 0.530], whereas the main effect of stimulation was not significant [*F*_(1, 19)_ = 2.180, *p* = 0.156, η^2^_*p*_ = 0.103]. There was also significant interactions between saccade type and probability [*F*_(1, 19)_ = 76.017, *p* < 0.001], stimulation and saccade type [*F*_(1, 19)_ = 8.026, *p* = 0.011, η^2^_*p*_ = 0.297], stimulation and probability [*F*_(1, 19)_ = 5.922, *p* = 0.025, η^2^_*p*_ = 0.238], and no 3-way interaction [*F*_(1, 19)_ = 0.275, *p* = 0.606, η^2^_*p*_ = 0.014].

The effect of probability was significant in both prosaccades [*t*_(19)_ = 5.979, *p* < 0.001] and antisaccades [*t*_(19)_ = −4.835, *p* < 0.001]. There was a significant RT difference between pro- and anti-saccades in the high probability locations [*t*_(19)_ = −7.392, *p* < 0.001], but no difference in the low probability locations [*t*_(19)_ = −1.458, *p* = 0.161]. To test Kanai et al.'s 2012 findings, we also compared prosaccdes to the low probability locations with and without tDCS, and observed a significant faster RT after anodal rFEF tDCS. This is consistent with Kanai et al.'s findings of faster prosaccades to low probably locations after anodal tDCS.

In the sham condition, *post-hoc* analysis revealed a significant effect of probability in prosaccades [*t*_(19)_ = 6.124, *p* < 0.001] and not antisaccades [*t*_(19)_ = −0.590, *p* = 0.562]. The RT difference between prosaccades and antisaccades was significant only in the high-probability locations [*t*_(19)_ = −6.987, *p* < 0.001] but not in the low-probability locations [*t*_(19)_ = 0.845, *p* = 0.409].

### Cathodal FEF tDCS

**Figure 3:**
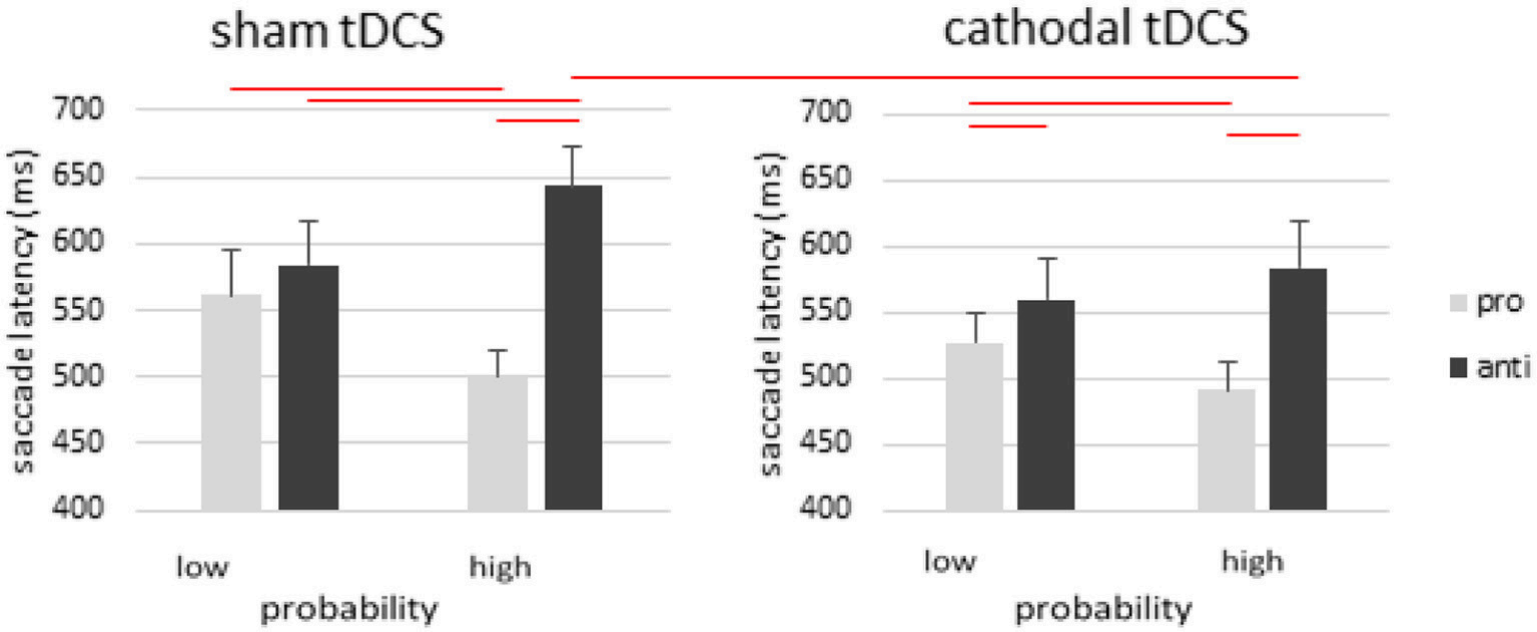
Effects of cathodal tDCS on rFEF. Cathodal tDCS facilitated pro- and anti-saccades in the low probability condition, but more so for the pro- than antisaccades, thereby showing a significant antisaccade cost in the low probability condition after tDCS. Importantly, cathodal tDCS produced faster antisaccade away from the high probability location such that the antisaccade cost is actually significantly reduced in the high probability condition after cathodal tDCS. Error bars represent standard error of the mean.

In cathodal rFEF tDCS group, the main effect of saccade type was significant [*F*_(1, 14)_ = 27.799, *p* < 0.001, η^2^_*p*_ = 0.665], whereas the effect of stimulation [*F*_(1, 14)_ = 3.381, *p* = 0.087, η^2^_*p*_ = 0.195] and probability were not significant [*F*_(1, 14)_ = 0.151, *p* = 0.703]. There was a significant 2-way interaction between stimulation and saccade type [*F*_(1, 14)_ = 5.569, *p* = 0.033, η^2^_*p*_ = 0.285], and between saccade type and probability [*F*_(1, 14)_ = 32.195, *p* < 0.001, η^2^_*p*_ = 0.697]. We also observed a significant 3-way interaction [*F*_(1, 14)_ = 0.275, *p* = 0.020, η^2^_*p*_ = 0.330]. The only non-significant interaction was the one between stimulation and probability [*F*_(1, 14)_ = 0.149, *p* = 0.705, η^2^_*p*_ = 0.011] (Figure 3).

To explore these interactions, the effect of probability for prosaccades was significant [prosaccade: *t*_(14)_ = 3.092, *p* = 0.008; antisaccade: *t*_(14)_ = −1.816, *p* = 0.091]. There was a significant RT difference between pro- and anti-saccades in low and high probability locations [high probability: *t*_(14)_ = −5.851, *p* < 0.001; low probability: *t*_(14)_ = −2.203, *p* = 0.045]. The effect of cathodal tDCS showed that the saccade latencies after cathodal rFEF tDCS was shorter only for antisaccades to high probability location [*t*_(14)_ = 2.436, *p* = 0.029].

In the sham-tDCS condition, *post-hoc* analysis revealed a significant effect of probability in prosaccade [*t*_(14)_ = 3.318, *p* = 0.005] and antisaccade trials [*t*_(14)_ = −3.668, *p* = 0.003]. The RT difference between prosaccades and antisaccades was significant only in the high-probability locations [*t*_(14)_ = −6.344, *p* < 0.001] but not in the low-probability locations [*t*_(14)_ = −1.441, *p* = 0.172].

**Figure 4:**
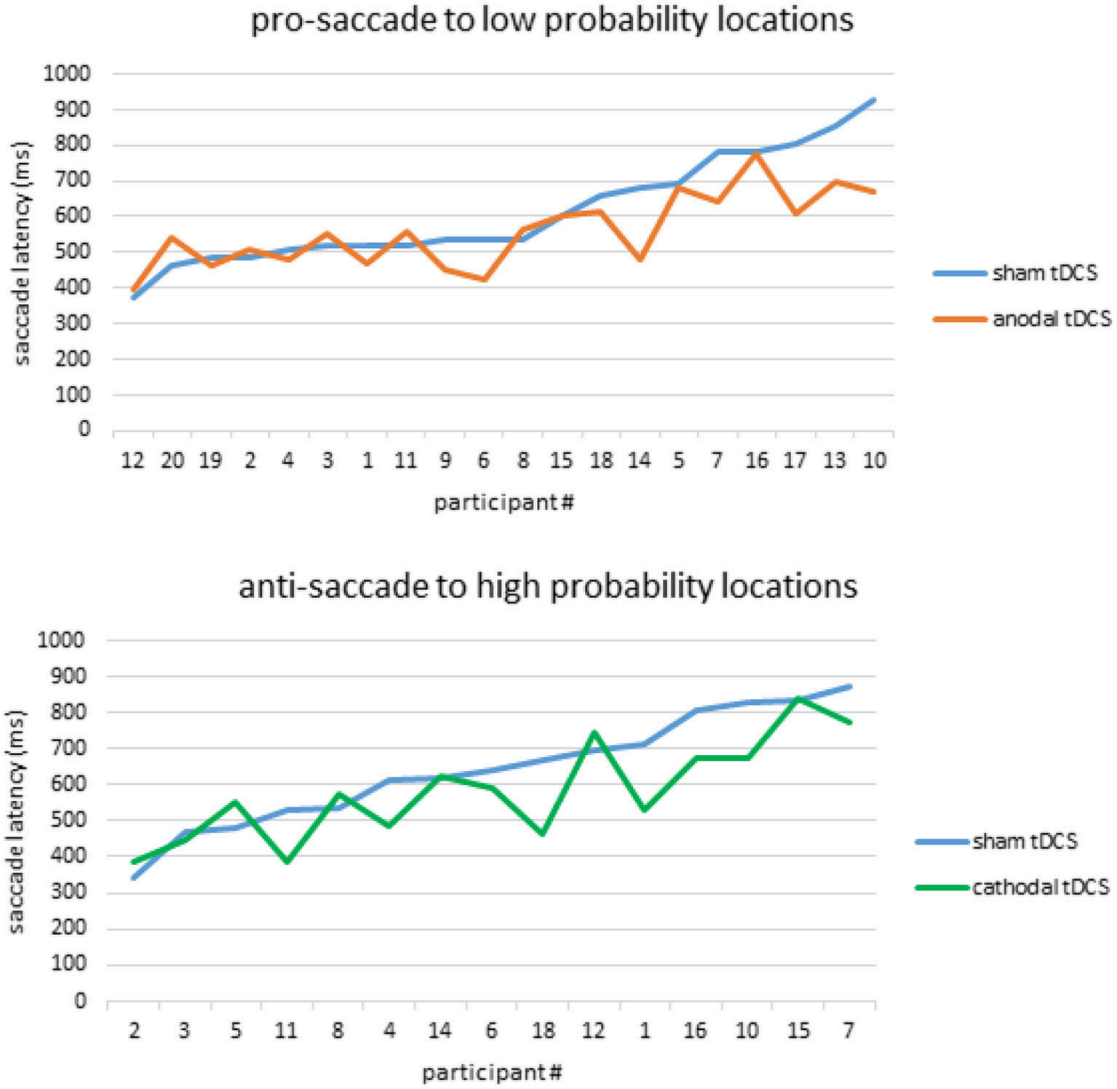
Individual differences in responsiveness to tDCS. Anodal tDCS facilitated prosaccades toward high-probability locations **(Top)** while cathodal tDCS facilitated antisaccades toward high-probability locations **(Bottom)**. These effects were mainly driven by low-performers who showed slower saccade latencies in the sham condition, as evidenced by the enlarged gap between the blue and orange lines in panel 1, and blue and green lines in panel 3.

Lastly, since individual variability in responsiveness to tDCS has been reported in many domains of cognitive functioning (e.g., Hsu et al., 2016; Tseng et al., 2018), we plotted all participants' SSRT from the sham condition against their own SSRT from the tDCS condition (Figure 4). As illustrated in Figure 4, the effect of anodal tDCS in facilitating prosaccades to the low-probablity locations, as well as the effect of cathodal tDCS in facilitating antisaccades to high-probability locations, seem to be mostly driven by the improvement from low-performers. High-performers, on the other hand, did not respond well to anodal or cathodal stimulation since their RT was already faster than their low-performing counterparts. This pattern of tDCS facilitation in low-performers has been well-documented in the literature of visual memory (e.g., see Juan et al., 2017, for a review), and here we report similar patterns in oculomotor performance.

### Control experiment (Anodal tDCS over SEF)

To ensure that the effects reported above were specific to FEF and not a general result of electricity flowing through the brain, in this control experiment we applied anodal tDCS over the SEF of a separate group of participants. Everything else was identical to the FEF experiment. Mean percent correct of total trials in control and tDCS conditions were 74.9% (pro = 78.4%, anti = 71.4%) and 73.9% (pro = 77.4%, anti = 70.2%).

**Figure 5:**
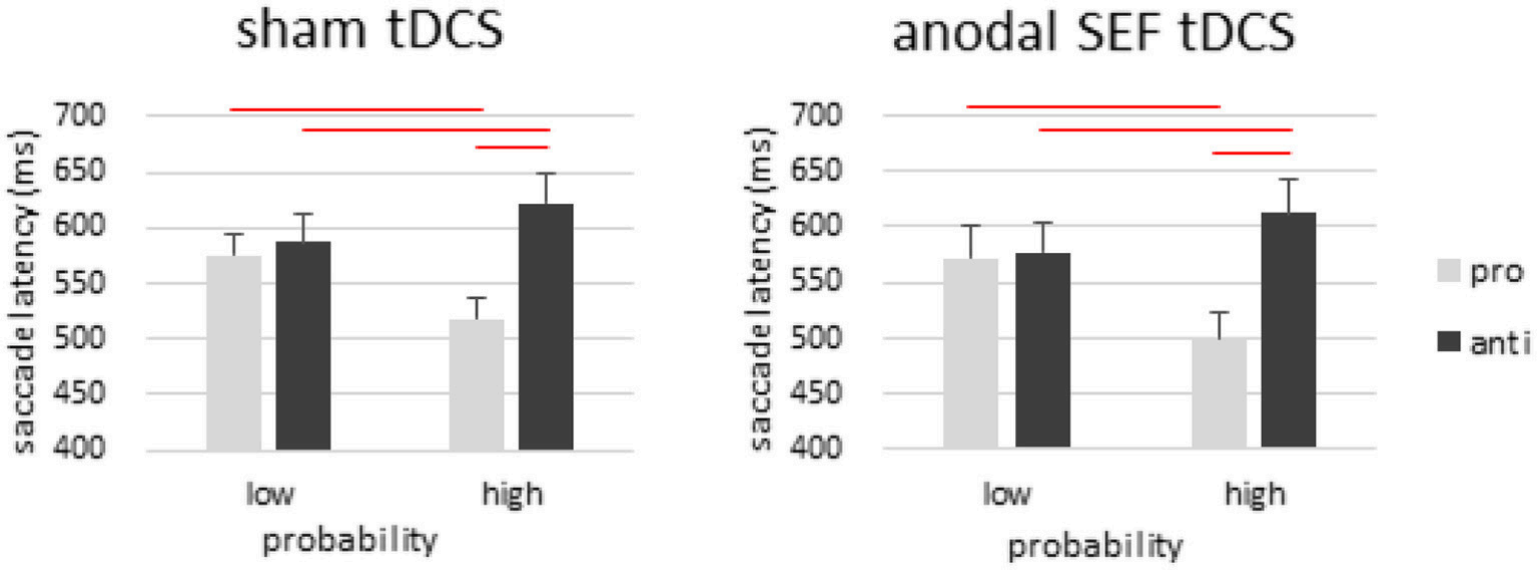
Results from the SEF control experiment. Anodal tDCS was applied over the SEF to control for the effect of electric current in the brain. There was a strong effect of probability with and without tDCS, and anodal tDCS did not change the pattern of any results from the sham condition. These results suggest that the probability task used here is quite specific to FEF functioning, and that the results we observed from FEF tDCS cannot be attributed to a general effect of electric current in any brain region. Error bars represent standard error of the mean.

For SRT data, three-way repeated ANOVA was performed with within-subject factors of stimulation, saccade type and probability. There was a significant effect of saccade type [*F*_(1, 17)_ = 28.839, *p* < 0.001, η^2^_*p*_ = 0.629], but no significant effect of stimulation [*F*_(1, 17)_ < 1, *p* > 0.05, η^2^_*p*_ = 0.020] or probability [*F*_(1, 17)_ = 3.208, *p* > 0.05, η^2^_*p*_ = 0.159]. We also observed a significant interaction between saccade type and probability [*F*_(1, 17)_ = 51.748, *p* < 0.001, η^2^_*p*_ = 0.753], whereas all other interactions were not statistically significant (Figure 5).

In antisaccade trials, we only observed a significant main effect of probability [*F*_(1, 17)_ = 11.381, *p* < 0.001, η^2^_*p*_ = 0.401]. Antisaccade latencies were longer in the high-probability location than in low-probability location. Post-hoc analysis showed that the effect of probability was not only present in prosaccade trials but also in antisaccade trials [sham: *t*_(17)_ = 3.0, *p* < 0.01; tDCS: *t*_(17)_ = 3.1, *p* < 0.01]. There was no significant main effect of tDCS [*F*_(1, 17)_ < 1, *p* > 0.05, η^2^_*p*_ = 0.014] or its interaction with location probability [antisaccade: *F*_(1, 17)_ < 1, *p* > 0.05, η^2^_*p*_ < 0.001].

## General discussion

In the present study we tested how anodal and cathodal tDCS over rFEF would impact the processing of spatial probability in both pro- and anti-saccades. We found that, regardless of tDCS, there was a strong effect of location probability in prosaccades, which was coupled with a slowing effect in antisaccades. When anodal tDCS was applied over rFEF, prosaccade to low-probability locations became faster. And with cathodal tDCS, antisaccades away from the high-probability cue location (in prosaccade trials) became faster. These observed effects were specific to rFEF because anodal tDCS over the SEF (i.e., control experiment) did not yield similar patterns. These results imply a substantial difference between the effects of anodal and cathodal tDCS over rFEF on the processing of probabilistic information, but not in a way that is consistent with the conventional assumption of tDCS bidrectionality. Furthermore, the effects of tDCS seemed to be driven by participants with slower SRT (i.e., low-performers).

### Prosaccade and antisaccade tradeoff

We first start with no-tDCS observations from the sham sessions. Looking at the results from the sham sessions alone, it is quite clear that the effect of spatial probability is strong in prosaccades. As such, although previous studies have found that antisaccade cost in RT can be eliminated when peripheral orienting and cue discrimination is moved to the center location (Liu et al., 2010; Chiau et al., 2011), in the present study we were able to reinstate the antisaccade cost by introducing high spatial location probabilities to the prosaccades. These results are consistent with previous reports of a robust spatial probability effect in oculomotor programming.

One observation is that in the high probability condition, where prosaccades enjoyed a much faster RT, there seemed to be a tradeoff in antisaccades such that antisaccades actually became *slower* when the cue was presented in the high probability location (Figures 2, 3, left). Upon closer inspection, this probability-induced tradeoff was also present in one of our earlier studies, although it was not noticed or discussed before (Liu et al., 2010, Experiment 2 and Figure 4). This tradeoff possibly suggest an automatic anticipation response toward the high probability location, which was accrued over the prosaccade trials and carried over to the antisaccade trials. This pro- and anti-saccade tradeoff in the high probability condition was only absent in the sham condition of Experiment 1, yet resurfaced after anodal tDCS was applied over the rFEF. Therefore, this tradeoff effect that is usually induced via probability, can also be induced via anodal tDCS over the rFEF. We think this suggests that spatial information was learned or encoded better with anodal tDCS applied over the rFEF. Furthermore, the fact that this slowing is specific to the high-probability cue locations, as opposed to all antisaccade trials in general, implies that rFEF activity is specifically relevant for processing probabilistic information, or the weighting of spatial locations, instead of domain-general oculomotor programming alone. Consequently, when a cue appeared at the probabilistically salient location, a reflexive response to that location is triggered, thus making an antisaccade to the opposite location more difficult and slower. This suggests an important role for rFEF in the encoding and processing of probabilistic information in visual space.

Since our task informed the participants of trial type at the start of every trial, we speculate that such anticipation to be a result of implicitly-learned (and transferred) association, rather than a reflection of deliberate decision-making. This would be quite consistent with the wealth of implicit learning literatures (Chun and Jiang, 1998; Fiser and Aslin, 2001; Tseng et al., 2011, 2012), and also in line with the fact that most of our participants did not realize the probability manipulation despite the seemingly-obvious contrast in location ratios.

### Anodal tDCS effect in prosaccades

Our anodal tDCS results showed a significant decrease in latencies for prosaccades to the low probability locations. This finding is somewhat similar to the Kanai et al. (2012) study that reported faster prosaccades and reduced saccade error rate to contralateral locations of the stimulated FEF. Although location probability was not manipulated in that particular study, their evenly-distributed probabilities can perhaps be equated with the low-probability locations from the current study since the three low-probability locations were also evenly distributed in terms of their probability ratio. Therefore, our anodal tDCS results seem to suggest that enhanced rFEF activity can also facilitate processing of the low probability locations.

The simplest mechanistic explanation for this effect is perhaps the activity level of rFEF—where high probability prosaccades have already enjoyed heightened activities from spatial probability, the low probability locations that lack probabilistic advantages can only be facilitated via tDCS. If we assume similar behavioral effect and neuronal mechanisms behind the facilitatory effect of spatial probability and anodal tDCS, as we have argued above, then it is plausible that the same neural populations that are already tuned to high probability locations cannot receive a further boost in activity via anodal tDCS. Meanwhile, on the other hand, neural assemblies that are tuned to low probability locations still have much room for increasing activity. This interpretation would be consistent with our understanding of saccade latency as dependent on the time for FEF to reach its threshold (Hanes and Schall, 1996), which is also true at the macroscopic level when we look at individual differences in responsiveness to tDCS. For example, in the visual working memory literature, it has been consistently demonstrated that high-performers can hit their cognitive ceiling (note: not task ceiling) in the sham condition, and therefore tDCS tend to be non-effective in these participants (e.g., Tseng et al., 2016, 2018). Indeed, this is also what we have observed here in our data: tDCS effect was mainly driven by those with slower SRT in the sham condition (Figure 4, top). This is discussed in more details in the tDCS section below.

One alternative explanation is that anodal tDCS may have modulated the processing of infrequent location information. This is based on the studies that suggest an important role for rFEF in processing unanticipated target locations. One study by Doricchi et al. (2010) employed a cuing paradigm to clarify the neural correlates of spatial and expectancy components of endogenous and stimulus-driven orienting of attention. They manipulated cue validity and probability, and found that regions in the right hemisphere such as the temporal-parietal junction (TPJ), superior parietal lobule (SPL), and FEF showed increased BOLD responses when targets appeared at unattended locations after a spatial expectation was established by a cue. In addition, another fMRI study by Shulman et al. (2009) manipulated cue types (shifting cue vs. maintaining cue) and the proportion of these two cue types while measuring BOLD signals, and found greater dorsal frontoparietal (including rFEF) activations when such reorienting was unexpected. In the current study, participants over time had formed an expectation of cues appearing in the high-probability location due to prior experience, which makes low-probability locations less anticipated and harder to reorient to.

### Cathodal tDCS effect in antisaccades

The results from the cathodal condition looks similar to those from the anodal condition, with two notable exceptions: (1) a significant antisaccade cost in the low-probability condition, and (2) faster antisaccades away from the high probability location.

The emerging antisaccade cost in the low-probability condition can be a result of faster prosaccade or slower antisaccade. From our results it seems that both saccade types were facilitated by cathodal tDCS over rFEF, but to a lesser extent in antisaccades. This presents a stark contrast to the results from Kanai et al. (2012), who found slower antisaccades after cathodal FEF tDCS. However, Kanai et al. attributed such slowing in antisaccades to a poor suppression of enhanced reflexive prosaccades to the cue. To this end, our data actually support their interpretation. That is, the surfacing of the antisaccade cost was in part due to faster prosaccade that trumped the improvement in antisaccade (Figure 3). Therefore, our cathodal tDCS results provide empirical support to their poor-suppression account.

The cathodal effect becomes quite different in the high probability condition. We observed a significant improvement in antisaccade latency from sham to cathodal tDCS condition. This effect is harder to account for, but seems to suggest that cathodal tDCS has produced an endpoint-specific effect. That is, if we look at the pro- and anti-saccade results together, the anodal and cathodal effects are actually coming from the same endpoint location. This is because low-probability endpoints in prosaccade trials happen to coincide with high-probability antisaccade endpoints. For instance, given that the high probability cue location is on the right side, anodal tDCS would facilitate prosaccades to the left side (i.e., low probability). In an antisaccade trial, if a high probability cue appears again (on the right), then the correct endpoint location would also be at the left spot. Therefore, our results imply that tDCS over rFEF seems to be endpoint-specific and favors the contralateral field. This is also consistent with the conclusion from Kanai et al. (2012).

### Anodal vs. cathodal tDCS in rFEF

One important observation of the present study is the dissociation between tDCS polarity and their effects on saccade type. That is, anodal stimulation seems to be more effective in prosaccades, and cathodal in antisaccades. If we insist on the anode-excitatory and cathode-inhibitory approach, then one possibility is that cathodal tDCS may have impaired reflexive prosaccades to the high probability location, thereby making the competing antisaccades away from that location faster. However, this interpretation is not supported by our prosaccade data, since this would also predict a tDCS-induced slowing in prosaccades in the cathodal condition. In fact, one benefit of the current paradigm is that we have already conducted a rTMS study using this task for comparison with the present findings (Liu et al., 2011). In that particular study, continuous theta burst TMS that is known to interfere cortical activity was applied over rFEF, and prosaccades to high probability location was indeed significantly impaired and prolonged (Liu et al., 2011). This rTMS finding is exactly what the impairment account would predict, but is actually in the opposite direction of what we have observed in the current study. Therefore, the actual effect of cathodal tDCS from the present study is incompatible with the notion of an impairment or inhibitory effect, and cathodal tDCS is clearly not an electrical equivalent of continuous theta burst TMS. On the other hand, our data from the anodal condition is more in line with the facilitation account, and looks more like the opposite effect of the rTMS study (Liu et al., 2011).

The results from the present study also suggest that tDCS models based on data from the motor cortex might need more fine-tuning when it comes to predicting activities in the FEF. Although early work from the 1960s in rats have established anodal and cathodal stimulation as excitatory and inhibitory (Albert, 1966; see Utz et al., 2010, for a review), this concept of a bidirectional neuro-modulatory effect of anodal and cathodal tDCS has been challenged recently (Jacobson et al., 2012; Krause et al., 2013; Pirulli et al., 2014; Hsu et al., 2016), and is also not supported by our data. This limitation may be due to differences in neurophysiology, anatomical connections, or even cognitive functioning (Juan et al., 2017). This also implies that, for every brain region, systematic investigations using precise tasks that target their activities are necessary in order to establish the effect of anodal and cathodal tDCS in these regions. In this light, it is important to note that our observation of an endpoint-specific effect may also be specific to tDCS over rFEF, and in the context of the current orienting paradigm.

### Individual differences in responsiveness to tDCS

Although anodal and cathodal tDCS had dissociable effects in different saccade types, one common observation across the anodal and cathodal conditions is the pattern of inter-individual variation in our participants' responsiveness to tDCS. Looking at Figure 4, it is quite clear that the decrease in SRT was coming from participants that had slower SRT in the sham condition. This was true for both the anodal and cathodal condition in prosaccade and antisaccade RT, respectively. This “room for improvement” pattern strongly resembles the patterns of tDCS effect in other studies, particularly the visual memory literature (e.g., Juan et al., 2017). For example, we have previously reported that anodal tDCS can interact with individual participant's natural baseline performance such that only the low-performers (i.e., below the median) would show an improvement effect from anodal tDCS, whereas the high-performers would show no effect at all (Tseng et al., 2012, 2016, 2018; Hsu et al., 2014). This would be consistent with our understanding of tDCS and its effect on firing rates (e.g., Nitsche et al., 2009) and can be well explained by the recently-proposed inhibition-excitation balance model (Krause et al., 2013), where overly-excited high-performers cannot improve their cognitive performance with anodal tDCS (for an excellent review on different models of tDCS mechanisms, see Fertonani and Miniussi, 2017). In our previous studies we have also reported that the magnitude of the anodal tDCS effect in these low-performers is usually somewhere between their natural baseline and below the high-performers. That is, even when tDCS is facilitative, it is not enough to bring them to the same level as their high-performing counterparts (Juan et al., 2017). This pattern is also true here at the task level, where tDCS did not bring down low-probability prosaccade latencies to the same level as high-probability did. This is plausible given that tDCS is applied non-selectively over all neurons within rFEF (as opposed to functional selection) and that only a small portion of the electricity can actually get through the skull (Opitz et al., 2016). It may be possible for future studies to direct currents to particular neural assemblies within FEF by applying tDCS while participants are actively performing a prosaccade task, thereby varying the threshold of different neurons to achieve state-dependent and targeted stimulation (Silvanto et al., 2008).

## Conclusion

In the present study we observed different effects of anodal and cathodal stimulation on pro- and anti-saccades, respectively. It was found that anodal tDCS over rFEF facilitated prosaccdes to low probability locations, whereas cathodal tDCS facilitated antisaccades away from the high probability cue location. Our control experiment also rules out SEF for a causal role in processing location probability since anodal tDCS over SEF did not yield any findings. Our findings on the effect of anodal tDCS on prosaccades are consistent with the anode-excitatory idea, and the observation that only low-performing participants and low-probability locations were facilitated closely resemble the state-dependent (Silvanto et al., 2008) and homeostatic (Krause et al., 2013) nature of tDCS that has been proposed by others (Juan et al., 2017; Silvanto and Cattaneo, 2017). The effect of cathodal tDCS on antisaccades away from high-probability locations is less conclusive, and possibly reflect an endpoint-selective mechanism within rFEF. Furthermore, these results suggest that the effect of anodal rFEF tDCS, as well as its interaction with baseline activity, is more generalizable and predictable across brain regions and different studies; while a comparison between the present cathodal results and our previous rTMS study suggest that cathodal rFEF tDCS is not an electrical equivalent of continuous theta burst TMS in the context of visual attention and oculomotor control.

## Author contributions

PT and C-HJ designed the study, conducted the experiment, analyzed data, and wrote the manuscript. M-CW conducted the experiment, analyzed data, and wrote the manuscript. Y-HL analyzed data and wrote the manuscript.

### Conflict of interest statement

The authors declare that the research was conducted in the absence of any commercial or financial relationships that could be construed as a potential conflict of interest. The reviewer AF and handling Editor declared their shared affiliation.

## References

[B1] Albert D. J. (1966). The effect of spreading depression on the consolidation of learning. Neuropsychologia 4, 49–64. 10.1016/0028-3932(66)90020-0

[B2] Allman J. M. (2000). Evolving Brains. New York, NY: WH Freeman.

[B3] Amador N. Schlag-Rey M. Schlag J. (2000). Reward-predicting and reward-detecting neuronal activity in the primarte supplementary eye field. J. Neurophysiol. 84, 2166–2170. 10.1152/jn.2000.84.4.2166 11024104

[B4] Antal A. Alekseichuk I. Bikson M. Brockmöller J. Brunoni A. R. Chen R.. (2017). Low intensity transcranial electric stimulation: safety, ethical, legal regulatory and application guidelines. Clin. Neurophysiol. 128, 1774–1809. 10.1016/j.clinph.2017.06.001 28709880 PMC5985830

[B5] Basso M. A. Wurtz R. H. (1997). Modulation of neuronal activity by target uncertainty. Nature 389, 66–69. 10.1038/37975 9288967

[B6] Bichot N. P. Schall J. D. (1999). Effects of similarity and history on neural mechanisms of visual selection. Nat. Neurosci. 2, 549–554. 10.1038/9205 10448220

[B7] Bikson M. Grossman P. Thomas C. Zannou A. L. Jiang J. Adnan T.. (2016). Safety of transcranial direct current stimulation: evidence based update 2016. Brain Stimul. 9, 641–661. 10.1016/j.brs.2016.06.004 27372845 PMC5007190

[B8] Bikson M. Grossman P. Zannou A. L. Kronberg G. Truong D. Boggio P.. (2017). Response to letter to the editor: safety of transcranial direct current stimulation: evidence based update 2016. Brain Stimul. 10, 986–987. 10.1016/j.brs.2017.06.007 28734680 PMC5975364

[B9] Carpenter R. H. (1999). Visual selection: neurons that make up their minds. Curr. Biol. 9, R595–R598. 10.1016/S0960-9822(99)80382-0 10469581

[B10] Carpenter R. H. Williams M. L. (1995). Neural computation of log likelihood in control of saccadic eye movements. Nature 377, 59–62. 10.1038/377059a0 7659161

[B11] Chen L. L. Wise S. P. (1995). Neuronal activity in the supplementary eye field during acquisition of conditional oculomotor associations. J. Neurophysiol. 73, 1101–1121. 10.1152/jn.1995.73.3.1101 7608758

[B12] Chiau H. Y. Tseng P. Su J. H. Tzeng O. J. Hung D. L. Muggleton N. G.. (2011). Trial type probability modulates the cost of antisaccades. J. Neurophysiol. 106, 515–526. 10.1152/jn.00399.2010 21543748 PMC3154828

[B13] Chun M. M. Jiang Y. (1998). Contextual cueing: implicit learning and memory of visual context guides spatial attention. Cogn. Psychol. 36, 28–71. 10.1006/cogp.1998.0681 9679076

[B14] Doricchi F. Macci E. Silvetti M. Macaluso E. (2010). Neural correlates of the spatial and expectancy components of endogenous and stimulus-driven orienting of attention in the Posner task. Cereb. Cortex 20, 1574–1585. 10.1093/cercor/bhp215 19846472

[B15] Dorris M. C. Munoz D. P. (1998). Saccadic probability influences motor preparation signals and time to saccadic initiation. J. Neurosci. 18, 7015–7026. 10.1523/JNEUROSCI.18-17-07015.1998 9712670 PMC6792986

[B16] Everling S. Fischer B. (1998). The antisaccade: a review of basic research and clinical studies. Neuropsychologia 36, 885–899. 10.1016/S0028-3932(98)00020-7 9740362

[B17] Fertonani A. Miniussi C. (2017). Transcranial electrical stimulation: what we know and do not know about mechanisms. Neuroscientist 23, 109–123. 10.1177/1073858416631966 PMC5405830 26873962

[B18] Fiser J. Aslin R. N. (2001). Unsupervised statistical learning of higher-order spatial structures from visual scenes. Psychol. Sci. 12, 499–504. 10.1111/1467-9280.00392 11760138

[B19] Gegenfurtner K. R. (2016). The interaction between vision and eye movements. Perception 45, 1333–1357. 10.1177/0301006616657097 27383394

[B20] Geng J. J. Behrmann M. (2005). Spatial probability as an attentional cue in visual search. Percept. Psychophys. 67, 1252–1268. 10.3758/BF03193557 16502846

[B21] Gmeindl L. Rontal A. Reuter-Lorenz P. A. (2005). Strategic modulation of the fixation-offset effect: dissociable effects of target probability on prosaccades and antisaccades. Exp. Brain Res. 164, 194–204. 10.1007/s00221-005-2242-9 15924234

[B22] Hallett P. E. (1978). Primary and secondary saccades to goals defined by instructions. Vision Res. 18, 1279–1296. 10.1016/0042-6989(78)90218-3 726270

[B23] Hanes D. P. Schall J. D. (1996). Neural control of voluntary movement initiation. Science 274, 427–430. 10.1126/science.274.5286.427 8832893

[B24] Hsu T. Y. Juan C. H. Tseng P. (2016). Individual differences and state-dependent responses in transcranial direct current stimulation. Front. Hum. Neurosci. 10:643. 10.3389/fnhum.2016.00643 28066214 PMC5174116

[B25] Hsu T. Y. Tseng P. Liang W.-K. Cheng S.-K. Juan C. H. (2014). Transcranial direct current stimulation over right posterior parietal cortex change prestimulus alpha oscillation in visual short-term memory task. Neuroimage 98, 306–313. 10.1016/j.neuroimage.2014.04.069 24807400

[B26] Im C. H. Park J. H. Shim M. Chang W. H. Kim Y. H. (2012). Evaluation of local electric fields generated by transcranial direct current stimulation with an extracephalic reference electrode based on realistic 3D body modeling. Phys. Med. Biol. 57:2137. 10.1088/0031-9155/57/8/2137 22452936

[B27] Jacobson L. Koslowsky M. Lavidor M. (2012). tDCS polarity effects in motor and cognitive domains: a meta-analytical review. Exp. Brain Res. 216, 1–10. 10.1007/s00221-011-2891-9 21989847

[B28] Jóhannesson Ó. I. Haraldsson H. M. Kristjánsson Á. (2013). Modulation of antisaccade costs through manipulation of target-location probability: only under decisional uncertainty. Vision Res. 93, 62–73. 10.1016/j.visres.2013.10.010 24148874

[B29] Juan C. H. Muggleton N. G. Tzeng O. J. Hung D. L. Cowey A. Walsh V. (2008). Segregation of visual selection and saccades in human frontal eye fields. Cereb. Cortex 18, 2410–2415. 10.1093/cercor/bhn001 18326522 PMC2536703

[B30] Juan C. H. Shorter-Jacobi S. M. Schall J. D. (2004). Dissociation of spatial attention and saccade preparation. Proc. Natl. Acad. Sci. U.S.A. 101, 15541–15544. 10.1073/pnas.0403507101 15489272 PMC524443

[B31] Juan C. H. Tseng P. Hsu T. Y. (2017). Elucidating and modulating the neural correlates of visuospatial working memory via noninvasive brain stimulation. Curr. Dir. Psychol. Sci. 26, 165–173. 10.1177/096372141667709522973420

[B32] Kanai R. Muggleton N. Walsh V. (2012). Transcranial direct current stimulation of the frontal eye fields during pro-and antisaccade tasks. Front. Psychiatry 3:45. 10.3389/fpsyt.2012.00045 22590461 PMC3349084

[B33] Krause B. Márquez-Ruiz J. Cohen Kadosh R. (2013). The effect of transcranial direct current stimulation: a role for cortical excitation/inhibition balance? Front. Hum. Neurosci. 7:602. 10.3389/fnhum.2013.00602 24068995 PMC3781319

[B34] Kristjánsson Á. (2007). Saccade landing point selection and the competition account of pro- and antisaccade generation: the involvement of visual attention–a review. Scand. J. Psychol. 48, 97–113. 10.1111/j.1467-9450.2007.00537.x 17430363

[B35] Kristjánsson Á. Chen Y. Nakayama K. (2001). Less attention is more in the preparation of antisaccades, but not prosaccades. Nat. Neurosci. 4, 1037–1042. 10.1038/nn723 11547337

[B36] Kristjánsson Á. Vandenbroucke M. W. Driver J. (2004). When pros become cons for anti- versus prosaccades: factors with opposite or common effects on different saccade types. Exp. Brain Res. 155, 231–244. 10.1007/s00221-003-1717-9 14661119

[B37] Land M. F. McLeod P. (2000). From eye movements to actions: how batsmen hit the ball. Nat. Neurosci. 3:1340. 10.1038/81887 11100157

[B38] Liu C. L. Chiau H. Y. Tseng P. Hung D. L. Tzeng O. J. Muggleton N. G.. (2010). Antisaccade cost is modulated by contextual experience of location probability. J. Neurophysiol. 103, 1438–1447. 10.1152/jn.00815.2009 20032240 PMC2887634

[B39] Liu C. L. Tseng P. Chiau H. Y. Liang W. K. Hung D. L. Tzeng O. J.. (2011). The location probability effects of saccade reaction times are modulated in the frontal eye fields but not in the supplementary eye field. Cereb. Cortex 21, 1416–1425. 10.1093/cercor/bhq222 21060112

[B40] Miller J. (1988). Components of the location probability effect in visual search tasks. J. Exp. Psychol. 14, 453–471. 10.1037/0096-1523.14.3.453 2971773

[B41] Milstein D. M. Dorris M. C. (2007). The influence of expected value on saccadic preparation. J. Neurosci. 27, 4810–4818. 10.1523/JNEUROSCI.0577-07.2007 17475788 PMC6672101

[B42] Milstein D. M. Dorris M. C. (2011). The relationship between saccadic choice and reaction times with manipulations of target value. Front. Neurosci. 5:122. 10.3389/fnins.2011.00122 22028681 PMC3199542

[B43] Munoz D. P. Everling S. (2004). Look away: the anti-saccade task and the voluntary control of eye movement. Nat. Rev. Neurosci. 5, 218–228. 10.1038/nrn1345 14976521

[B44] Nitsche M. A. Boggio P. S. Fregni F. Pascual-Leone A. (2009). Treatment of depression with transcranial direct current stimulation (tDCS): a review. Exp. Neurol. 219, 14–19. 10.1016/j.expneurol.2009.03.038 19348793

[B45] Nitsche M. A. Fricke K. Henschke U. Schlitterlau A. Liebetanz D. Lang N.. (2003a). Pharmacological modulation of cortical excitability shifts induced by transcranial direct current stimulation in humans. J. Physiol. 553, 293–301. 10.1113/jphysiol.2003.049916 12949224 PMC2343495

[B46] Nitsche M. A. Liebetanz D. Lang N. Antal A. Tergau F. Paulus W. (2003b). Safety criteria for transcranial direct current stimulation in humans. Clin. Neurophysiol. 114, 2220–2222. 10.1016/S1388-2457(03)00235-9 14580622

[B47] Nitsche M. A. Paulus W. (2001). Sustained excitability elevations induced by transcranial DC motor cortex stimulation in humans. Neurology 57, 1899–1901. 10.1212/WNL.57.10.1899 11723286

[B48] Olk B. Kingstone A. (2003). Why are antisaccades slower than prosaccades? A novel finding using a new paradigm. Neuroreport 14, 151–155. 10.1097/00001756-200301200-00028 12544848

[B49] Opitz A. Falchier A. Yan C. G. Yeagle E. M. Linn G. S. Megevand P.. (2016). Spatiotemporal structure of intracranial electric fields induced by transcranial electric stimulation in humans and nonhuman primates. Sci. Rep. 6:31236. 10.1038/srep31236 27535462 PMC4989141

[B50] Peterson M. S. Kramer A. F. (2001). Attentional guidance of the eyes by contextual information and abrupt onsets. Percept. Psychophys. 63, 1239–1249. 10.3758/BF03194537 11766947

[B51] Pirulli C. Fertonani A. Miniussi C. (2014). Is neural hyperpolarization by cathodal stimulation always detrimental at the behavioral level? Front. Behav. Neurosci. 8:226. 10.3389/fnbeh.2014.00226 25018709 PMC4073198

[B52] Ro T. Farnè A. Chang E. (2002). Locating the human frontal eye fields with transcranial magnetic stimulation. J. Clin. Exp. Neuropsychol. 24, 930–940. 10.1076/jcen.24.7.930.8385 12647769

[B53] Schall J. D. (2001). Neural basis of deciding, choosing and acting. Nat. Rev. 2, 33–42. 10.1038/35049054 11253357

[B54] Schall J. D. (2004). On the role of frontal eye field in guiding attention and saccades. Vision Res. 44, 1453–1467. 10.1016/j.visres.2003.10.025 15066404

[B55] Schall J. D. (2009). Frontal eye field, in Encyclopedia of Neuroscience, ed Squire L. R. (Oxford: Academic), 367–374.

[B56] Schall J. D. Hanes D. P. (1993). Neural basis of saccade target selection in frontal eye field during visual search. Nature 366, 467–469. 10.1038/366467a0 8247155

[B57] Schall J. D. Thompson K. G. (1999). Neural selection and control of visually guided eye movements. Annu. Rev. Neurosci. 22, 241–259. 10.1146/annurev.neuro.22.1.241 10202539

[B58] Schiller P. H. Kendall J. (2004). Temporal factors in target selection with saccadic eye movements. Exp. Brain Res. 154, 154–159. 10.1007/s00221-003-1653-8 13680050

[B59] Schlag-Rey M. Amador N. Sanchez H. Schlag J. (1997). Antisaccade performance predicted by neuronal activity in the supplementary eye field. Nature 390, 398–401. 10.1038/37114 9389478

[B60] Shulman G. L. Astafiev S. V. Franke D. Pope D. L. Snyder A. Z. McAvoy M. P.. (2009). Interaction of stimulus-driven reorienting and expectation in ventral and dorsal frontoparietal and basal ganglia-cortical networks. J. Neurosci. 29, 4392–4407. 10.1523/JNEUROSCI.5609-08.2009 19357267 PMC2743562

[B61] Silvanto J. Cattaneo Z. (2017). Common framework for “virtual lesion” and state-dependent TMS: The facilitatory/suppressive range model of online TMS effects on behavior. Brain Cogn. 119, 32–38. 10.1016/j.bandc.2017.09.007 28963993 PMC5652969

[B62] Silvanto J. Muggleton N. Walsh V. (2008). State-dependency in brain stimulation studies of perception and cognition. Trends Cogn. Sci. 12, 447–454. 10.1016/j.tics.2008.09.004 18951833

[B63] Stagg C. J. Nitsche M. A. (2011). Physiological basis of transcranial direct current stimulation. Neuroscientist 17, 37–53. 10.1177/1073858410386614 21343407

[B64] Stuphorn V. Taylor T. L. Schall J. D. (2000). Performance monitoring by the supplementary eye field. Nature 408, 857–860. 10.1038/35048576 11130724

[B65] Summerfield C. Egner T. (2009). Expectation (and attention) in visual cognition. Trends Cogn. Sci. 13, 403–409. 10.1016/j.tics.2009.06.003 19716752

[B66] Thompson K. G. Bichot N. P. Schall J. D. (1997). Dissociation of target selection from saccade planning in macaque frontal eye field. J. Neurophysiol. 77, 1046–1050. 10.1152/jn.1997.77.2.1046 9065870

[B67] Thompson K. G. Hanes D. P. Bichot N. P. Schall J. D. (1996). Perceptual and motor processing stages identified in the activity of macaque frontal eye field neurons during visual search. J. Neurophysiol. 76, 4040–4055. 10.1152/jn.1996.76.6.4040 8985899

[B68] Tseng P. Chang Y. T. Liang W. K. Chang C. F. Juan C. H. (2016). The critical role of phase difference in gamma oscillation within the temporoparietal network for binding visual working memory. Sci. Rep. 6: 32138. 10.1038/srep32138 27573864 PMC5004173

[B69] Tseng P. Hsu T. Y. Chang C. F. Tzeng O. J. Hung D. L. Muggleton N. G.. (2012). Unleashing potential: transcranial direct current stimulation over the right posterior parietal cortex improves change detection in low-performing individuals. J. Neurosci. 32, 10554–10561. 10.1523/JNEUROSCI.0362-12.2012 22855805 PMC6621415

[B70] Tseng P. Hsu T. Y. Tzeng O. J. Hung D. L. Juan C. H. (2011). Probabilities in implicit learning. Perception 40, 822–829. 10.1068/p6833 22128554

[B71] Tseng P. Iu K. C. Juan C. H. (2018). The critical role of phase difference in theta oscillation between bilateral parietal cortices for visuospatial working memory. Sci. Rep. 8:349. 10.1038/s41598-017-18449-w 29321584 PMC5762658

[B72] Uchida Y. Lu X. Ohmae S. Takahashi T. Kitazawa S. (2007). Neuronal activity related to reward size and rewarded target position in primate supplementary eye field. J. Neurosci. 27, 13750–13755. 10.1523/JNEUROSCI.2693-07.2007 18077686 PMC6673633

[B73] Utz K. S. Dimova V. Oppenländer K. Kerkhoff G. (2010). Electrified minds: transcranial direct current stimulation (tDCS) and galvanic vestibular stimulation (GVS) as methods of non-invasive brain stimulation in neuropsychology—a review of current data and future implications. Neuropsychologia 48, 2789–2810. 10.1016/j.neuropsychologia.2010.06.002 20542047

