# Supplementary material for: Anodal and Cathodal tDCS Over the Right Frontal Eye Fields Impacts Spatial Probability Processing Differently in Pro- and Anti-saccades
Source: Front Neurosci. 2018 Jun 27;12:421. doi: 10.3389/fnins.2018.00421 (PMC6030360; doi:10.3389/fnins.2018.00421)
Supplement: Supplementary file 1 [file Table_1.pdf]

	Sham Pro Low	Sham Pro High	Sham Anti Low	Sham Anti High
%	70.63%	86.62%	74.38%	73.33%
SE	3.25%	2.23%	3.07%	3.84%
	Anodal Pro Low	Anodal Pro High	Anodal Anti Low	Anodal Anti High
%	67.71%	86.01%	77.64%	79.79%
SE	3.64%	1.41%	2.33%	2.55%
	Sham Pro Low	Sham Pro High	Sham Anti Low	Sham Anti High
%	74.44%	88.74%	73.70%	71.11%
SE	3.72%	1.82%	2.99%	4.46%
	Cathodal Pro Low	Cathodal Pro Hig	Cathodal Anti Low	Cathodal Anti High
%	73.89%	89.28%	74.72%	75.28%
SE	3.89%	1.45%	3.27%	4.12%

Supplementary Table 1. Percentage of correct trials across all conditions.
